# Gene network analysis identifies rumen epithelial cell proliferation, differentiation and metabolic pathways perturbed by diet and correlated with methane production

**DOI:** 10.1038/srep39022

**Published:** 2016-12-14

**Authors:** Ruidong Xiang, Jody McNally, Suzanne Rowe, Arjan Jonker, Cesar S. Pinares-Patino, V. Hutton Oddy, Phil E. Vercoe, John C. McEwan, Brian P. Dalrymple

**Affiliations:** 1 CSIRO Agriculture, Queensland Bioscience Precinct, 306 Carmody Rd, 4067 Queensland, Australia; 2 CSIRO Agriculture, FD McMaster Laboratory, Armidale, NSW 2350, Australia; 3 AgResearch Limited, Invermay Agricultural Centre, Mosgiel 9053, New Zealand; 4 AgResearch Limited, Grasslands Research Centre, Palmerston North 4442, New Zealand; 5 CSIRO Agriculture, Black Mountain Laboratories, Clunies Ross Street, 2601 ACT, Australia; 6 NSW Department of Primary Industries, Beef Industry Centre, University of New England, Armidale, NSW 2351, Australia; 7 School of Animal Biology and Institute of Agriculture, The University of Western Australia, 35 Stirling Highway, Crawley WA 6009, Australia

## Abstract

Ruminants obtain nutrients from microbial fermentation of plant material, primarily in their rumen, a multilayered forestomach. How the different layers of the rumen wall respond to diet and influence microbial fermentation, and how these process are regulated, is not well understood. Gene expression correlation networks were constructed from full thickness rumen wall transcriptomes of 24 sheep fed two different amounts and qualities of a forage and measured for methane production. The network contained two major negatively correlated gene sub-networks predominantly representing the epithelial and muscle layers of the rumen wall. Within the epithelium sub-network gene clusters representing lipid/oxo-acid metabolism, general metabolism and proliferating and differentiating cells were identified. The expression of cell cycle and metabolic genes was positively correlated with dry matter intake, ruminal short chain fatty acid concentrations and methane production. A weak correlation between lipid/oxo-acid metabolism genes and methane yield was observed. Feed consumption level explained the majority of gene expression variation, particularly for the cell cycle genes. Many known stratified epithelium transcription factors had significantly enriched targets in the epithelial gene clusters. The expression patterns of the transcription factors and their targets in proliferating and differentiating skin is mirrored in the rumen, suggesting conservation of regulatory systems.

The acquisition of nutrients in ruminants (including sheep, cattle, buffalo and goats) is the consequence of complex interactions between the diet, the rumen microbial population and the host. The plant material enters the rumen complex (a specialized forestomach) and is subjected to microbial fermentation. The microbial fermentation primarily produces short chain fatty acids (SCFAs), taken up from the rumen^1^, with CH_4_ as a by-product^2^. The estimated loss of feed gross energy as CH_4_ in cattle is up to 12%^3^. This CH_4_ also contributes to the approximately one third of agricultural CH_4_ emissions that are from livestock^4^, mainly the ruminants, and hence to global warming. The rumen wall is a stratified epithelium related to skin^5^ (Supplementary Figure S1) surrounded by a muscular layer which contracts to move and mix the rumen contents. The submucosa, a dense region of extracellular matrix, lies between the muscle and the lamina propria layer, which together with the epithelium forms the mucosa. The lamina propria accommodates active immune cells, loose extracellular matrix and blood vessels. Above the lamina propria are the epithelial layers transitioning from basal stem cells^6^, which amplify and differentiate^7^ undergoing cornification through keratinsation to form the internal surface of the rumen^8^, and eventually sloughing off the surface into the rumen contents^9^. The papillae lie on the interior surface of the rumen and contain mainly epithelial layers and lamina propria, and a small amount of submucosa and muscle^8^.

Previous studies have shown that rumen keratinsed epithelial growth^10^,^11^ and ruminal metabolism^12^ adapt to different diet conditions. Such adaptation may be associated with variation in rumen digestion efficiency and the mechanisms mediating host genetic control of rumen CH_4_ production via the rumen wall^13^,^14^,^15^. However, the exact molecular mechanisms by which the different layers of the rumen wall, including the epithelium, respond to diet and their possible relationships with CH_4_ production are unknown. To understand how the layers of rumen wall specifically respond to diet and influence CH_4_ production, we analyzed the transcriptome of full depth rumen wall samples from 24 female sheep fed different amounts and qualities of feed and phenotyped for a number of traits including CH_4_ production. Full depth rumen wall samples were chosen to enable us to study all layers of the rumen wall, avoid complex sample procedures to separate layers (minimizing sample variation introduced by sample processing) and to retain the context of relative gene expression in the different layers. Since the rumen shares a significant number of transcriptomic features with the skin^16^, a well understood epithelial system^17^, we analysed the rumen transcriptome in parallel with human skin datasets to further understand the regulatory mechanisms in the rumen epithelium.

## Results

### Assignment of gene sub-networks and clusters to rumen wall layers

**Figure 1:**
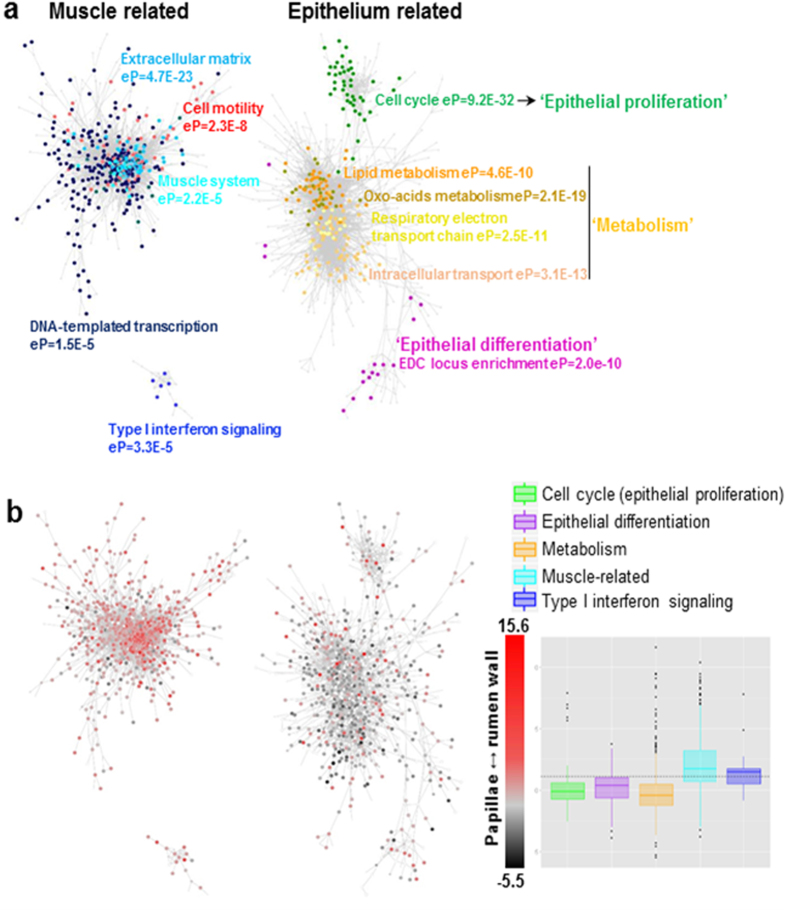
Sheep full thickness rumen wall gene expression correlation network. **(a)** The global network colored by GO-term enriched pathways with eP as corrected enrichment P values. Words in quotes are the names of the major gene clusters defined in the text. **(b)** The global network with the results of transcriptomic comparisons of the full thickness rumen wall vs. rumen papillae (P and FDR <0.05) mapped. The gradient from red to black represents log_2_ fold changes (absolute value >1) from high expression in the full thickness rumen wall to high expression in the rumen papillae. The distribution box plot of log_2_ fold changes of expression of identified gene clusters for each comparison is also shown.

A global gene expression correlation network filtered for gene-gene correlations with a coefficient of >+0.8 was generated from the 24 sheep full thickness rumen wall samples. The network node and edge information is in Supplementary Data S1. This network contained two major negatively correlated (average r = −0.65) sub-networks, apparently related to the muscle and the epithelial components of the wall of the rumen based on gene ontology (GO) analysis (Fig. 1a, Table 1, Supplementary Table S1). In the “epithelium” sub-network, the GO-terms related to cell cycle and metabolism were significantly enriched, as were epidermal differentiation complex (EDC) genes^5^,^16^ (Fig. 1a, Table 1). On the other hand, the “muscle” sub-network was significantly enriched for genes with the GO-terms, “extracellular matrix”, “cell motility”, “muscle system processes” and “DNA-templated transcription” (Fig. 1a, Table 1). In addition, a third small sub-network (average r = −0.22 with the muscle sub-network and average r = +0.28 with the epithelial sub-network) was significantly enriched for the GO-term “type 1 interferon signaling” (Fig. 1a, Table 1). To confirm the rumen layer(s) of origin of the subnetworks we compared the full thickness rumen wall transcriptome to bovine rumen papillae (enriched for rumen epithelium^18^) and mouse small intestinal smooth muscle transcriptome data^19^ (Fig. 1b). Overall, the comparisons supported the assignment of the “muscle” and “epithelium”-related sub-networks to the rumen muscle and epithelial layers, respectively (Table 1). As shown in Table 1, the proposed “muscle” sub-network was significantly (hypergeometric P, hP = 3.2E-74) enriched for genes showing higher expression in the full thickness rumen wall (contains muscle and papillae) than in rumen papillae (have very little or no muscle). In the proposed “epithelium” subnetwork, the cell cycle and metabolism clusters were significantly enriched for genes showing higher expression in rumen papillae than in the full thickness rumen wall. No enrichment of genes involved in epithelial differentiation for differential expression between the full thickness rumen wall and rumen papillae was expected as both full thickness rumen wall and rumen papillae contained keratinised layers. Consistent results were also obtained from gene expression comparisons between smooth muscle and full thickness rumen wall, and between smooth muscle and rumen papillae (Supplementary Figure S2). Genes involved in the type I interferon pathway appeared to always show higher expression in muscle-related than in epithelium-related layers (Fig. 1b, Supplementary Figure S2a,b).

**Figure 2:**
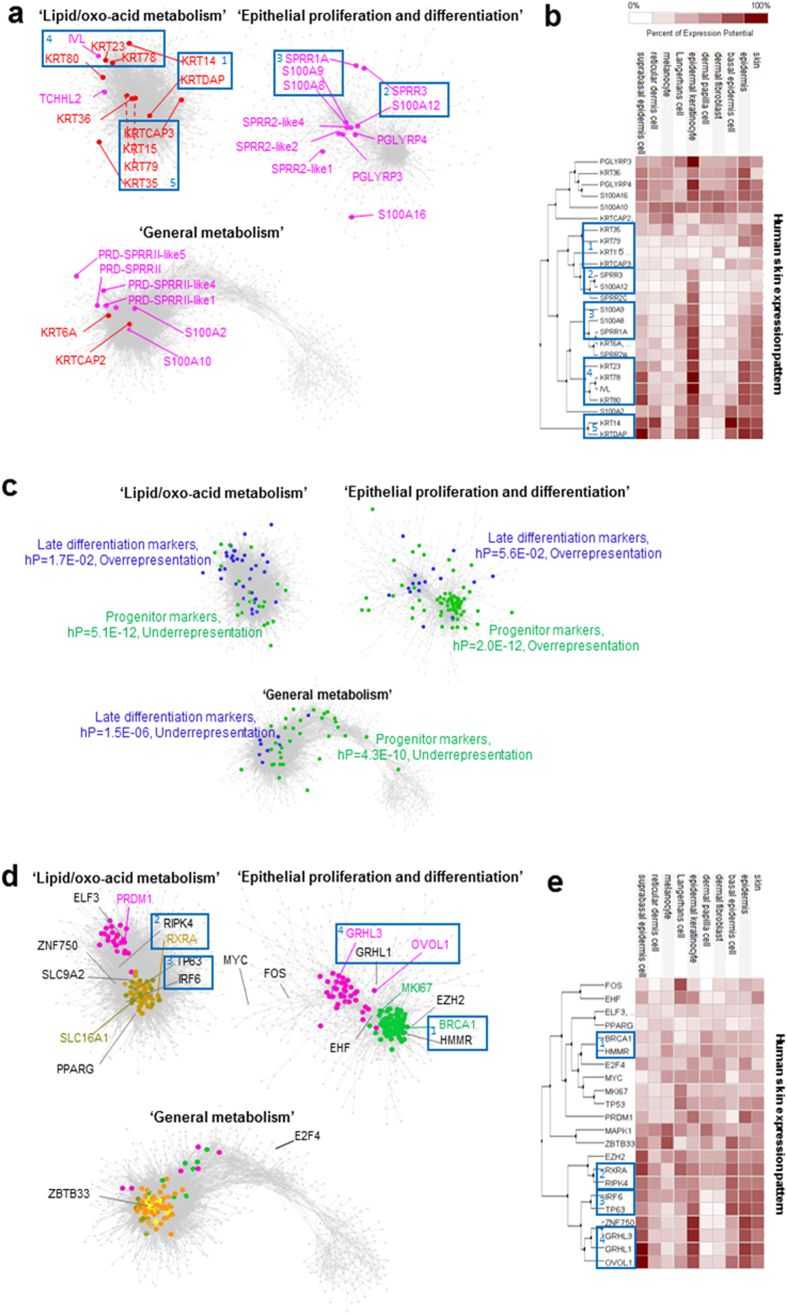
Expanded epithelium gene expression correlation network. (**a**) Locations of sheep EDC^5^ and keratin genes^16^ are indicated. (**b**) Clustering of expression of EDC and keratin genes in human skin, data from Genevestigator^54^. Percent of expression potential: normalised average relative to the top 1 percent values of corresponding probe set across all samples included in Genevestigator^54^. Genes with consistent expression patterns between full thickness rumen wall and skin are highlighted in boxes labelled with the same numbers. (**c**) Genes with enhanced expression in the progenitor (green), early (no overlap) and late (blue) differentiation stages of an *in vitro* keratinocyte differentiation model^17^ are indicated. (**d**) genes are coloured according to the membership of clusters in the global epithelium network (Fig. 1a): purple: ‘epithelial differentiation’; green: ‘cell cycle’ (epithelial proliferation); olive: ‘oxo-acids metabolism’: brown: ‘lipid metabolism’; yellow: ‘respiratory electron transport chain’; light orange: ‘intracellular transport’. Key transcription factors (Table 2) are labeled. Genes with consistent expression patterns between rumen and skin are highlighted in boxes labelled with the same numbers. (**e**) Clustering of expression of key transcription factors in human skin, data from Genevestigator. Percent of expression potential: normalised average relative to the top 1 percent values of corresponding probe set across all samples included in Genevestigator.

By removing the genes in the muscle- and interferon-related clusters, an expanded epithelium sub-network was constructed using less stringent cutoffs to further explore the epithelial gene clusters (Fig. 2). The network node and edge data is in Supplementary Data S2. Genes related to cell cycle and cornified epithelium in the global network were merged into one more tightly connected cluster in the expanded epithelium sub-network. In contrast, the global network identified ‘metabolism’ genes were further separated into two clusters in the expanded epithelium sub-network; one cluster containing lipid/oxo-acid genes was defined as ‘lipid/oxo-acid metabolism’ and the other containing respiratory electron transport chain and intracellular transport genes was defined as ‘general metabolism’. Further, based on Glay network clustering^20^, the ruminant-specific (and with highly rumen biased expression) *PRD-SPRRII* family members and *TCHHL2*^5^, EDC genes were grouped within the ‘general’ and ‘lipid/oxo-acid’ metabolism clusters respectively, whilst the expression of the conserved mammalian EDC *SPRR2, PGLYRP* and *S100A* family members lay within the cell cycle and cornified epithelium cluster (Fig. 2a). However, the majority of the keratin genes present in the network were located in the ‘lipid/oxo-acid metabolism’ cluster (Fig. 2a). Clustering of these genes based on their expression across cell types in human skin was similar to their clustering in the rumen wall (Fig. 2b).

To confirm the probable cellular origins of the gene expression signals the genes enriched in the progenitor, early differentiation and late differentiation stages of an *in vitro* model of epidermal keratinocyte differentiation^17^ were also mapped to the expanded rumen epithelium sub-network (Fig. 2c, Table 1). The sub-cluster enriched for cell cycle genes was also highly enriched for genes representing epidermal progenitor markers (hP = 2E-12), consistent with the expression of genes in this sub-cluster representing the cell division activity of the basal stem cells of the epithelium. The cell cycle gene enriched sub-cluster was defined as the ‘epithelial proliferation’ cluster. In contrast, the lipid/oxo-acid’ and ‘general metabolism’ clusters were significantly depleted for progenitor enriched genes (hP < 4.3E-10), probably reflecting the more general expression of metabolism genes across the cell types. Similarly genes enhanced in late differentiation were significantly under-represented in the ‘general metabolism’ cluster (hP = 1.5E-06). The ‘lipid/oxo-acid metabolism’ (hP = 1.7E-2) cluster had significant enrichment for late differentiation stage genes, but with localized clustering of overlapping genes (Fig. 2c). This is consistent with the presence of the EDC and keratin genes in these clusters as keratinisation is the last stage of epithelial differentiation.

### Identification of key transcription factors

**Figure 3:**
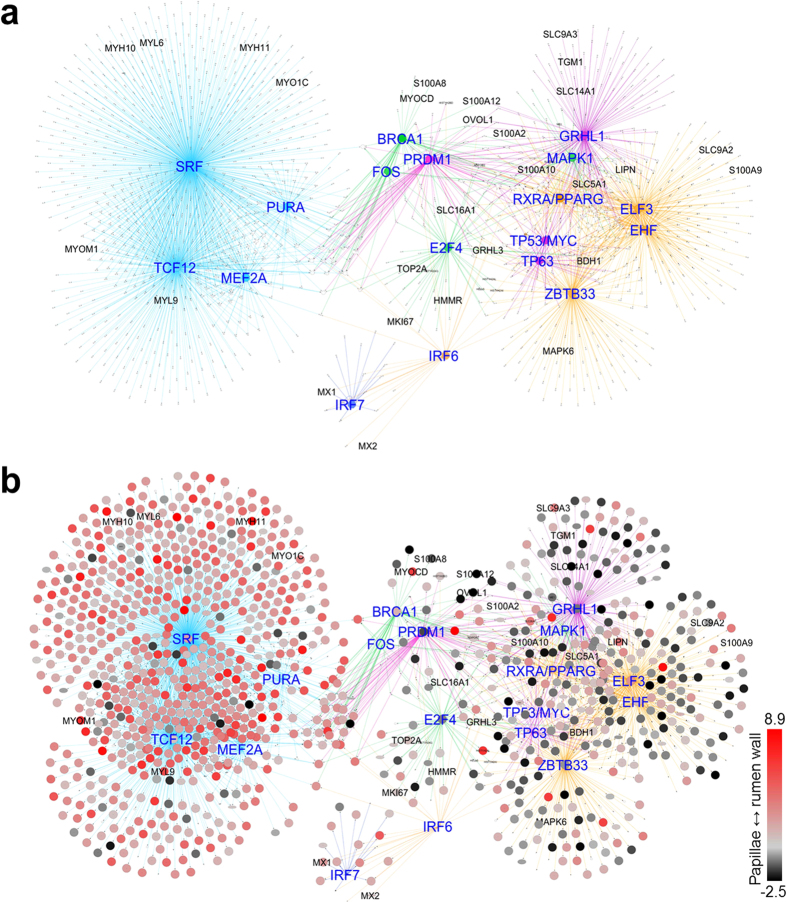
Network of transcription factors and predicted targets identified by iRegulon. **(a)** Network with edges coloured according to network structure shown in Fig. 1a. (**b**) Network with the results of transcriptomic comparisons of the full thickness rumen wall vs rumen papillae. The gradient from red to black represents log_2_ fold changes from high expression in the full thickness rumen wall to high expression in the rumen papillae.

To identify regulatory mechanisms in the different processes and layers of the rumen wall a network of transcription factors (TFs) and their targets was constructed using iRegulon (Fig. 3, Supplementary Table S1, S2, Supplementary Data S3). TFs with significant enrichment of predicted target genes in the global network and present in the global or expanded epithelial network, or known to be involved in regulation of epithelial or muscle development and function were identified (Table 2, Supplementary Table S2). In general, the expression status of TFs and their predicted targets in the full thickness rumen wall and rumen papillae comparison mirrored the results of the mRNA network analyses described above (Fig. 1b). The majority of TFs identified in the epithelial subnetwork (Table 2), and their targets, were more highly expressed in rumen papillae than in the full thickness rumen wall (Fig. 3b).

Members of the IRF-family had significantly enriched targets in the “interferon” cluster and in the epidermal sub-network. The presence of *IRF7* in the interferon cluster (Supplementary Table S1), and *IRF6* in the expanded epidermal sub-network (Fig. 2), suggests that these may be the active members of the family for the rumen epithelium (Table 2). The role of IRF7 in airway epithelium protection^21^ and the cooperation of IRF6 with RIPK4 as critical regulators of keratinocyte differentiation^22^ supports this conclusion.

The clustering of key epithelial TFs generally showed consistency between the rumen and human skin, providing evidence for the assignment of gene clusters to different layers and/or processes in the rumen (Table 2, Fig. 2d,e). For example, the TFs in the cell cycle cluster are preferentially expressed in basal cells from the skin (Table 2), whilst *GRHL1, GRHL3* and *OVOL1* are more highly expressed in differentiating cells in the suprabasal layers of the skin (Fig. 2e). Consistent with this they are clustered with the ‘epithelial proliferation-and-differentiation’ genes and the *SPRR2*–gene family (Fig. 2a), most highly expressed in terminally differentiating squamous cells in mouse skin^23^. Moreover, key TFs presented in the rumen ‘lipid/oxo-acid metabolism’ cluster including *TP63, IRF6, RIPK4* and *RXRA* were again grouped together based on human skin expression and showed high expression in suprabasal or basal epidermal layers (Fig. 2e).

### Relationships between functional gene sets, diet and ruminal parameters

**Figure 4:**
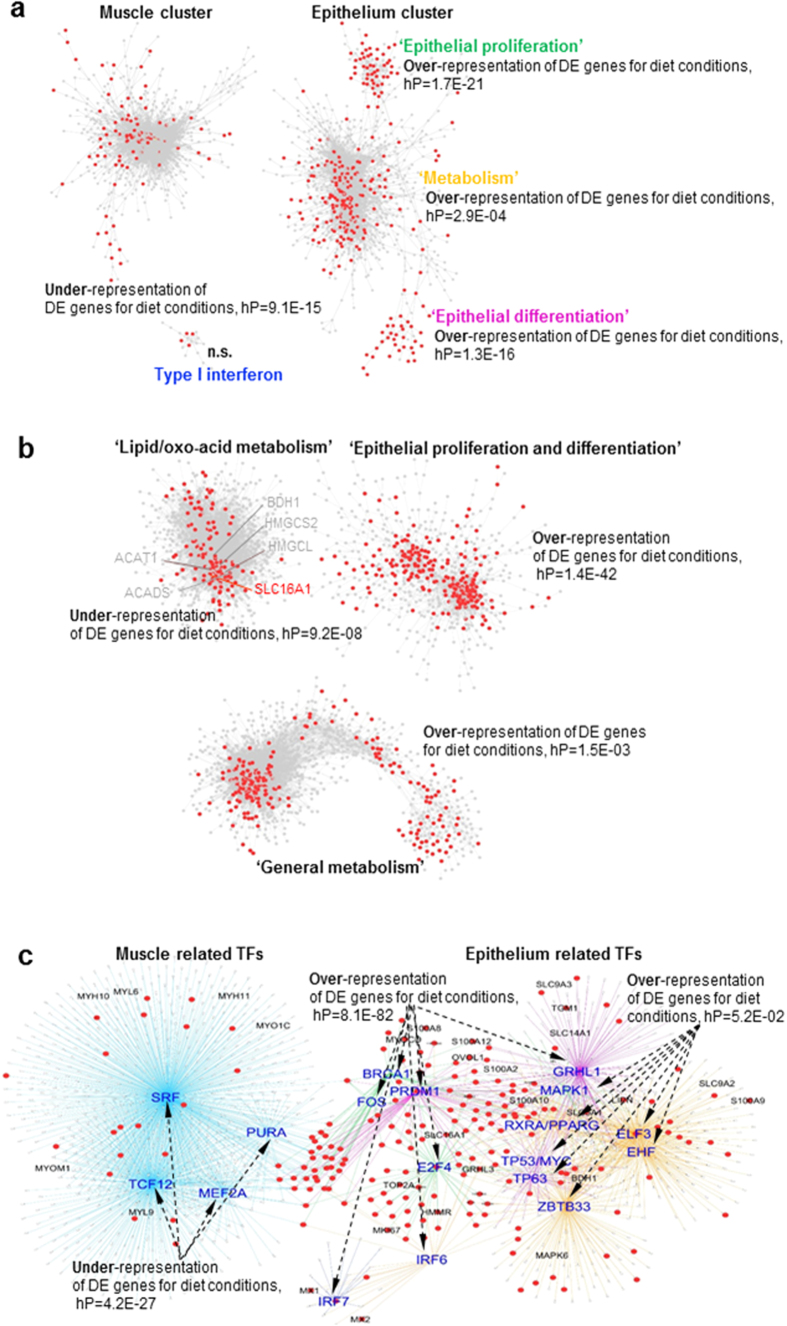
Differential expressed genes (P < 0.05, red dots) for overall effects of feed quality and level (ANOVA) in experimental design were mapped to the global network (**a**), the expanded epithelium network (**b**), and the network of transcription factors and predicted targets identified by iRegulon (**c**). Hypergeometric *P*-values (hP) indicate the representation of differentially expressed genes for diet conditions in the identified clusters. In (**b**) individual genes involved in the previously identified rumen ketone body metabolism pathway^16^ are labelled. In (**c**) dashed arrows point to hP for enrichment of differentially expressed genes in the cluster of transcription factor and its targets.

Genes showing significant differential expression (DE) (P < 0.05) based on overall effects (ANOVA) of feed levels and qualities were mapped to the high-stringency filtered global network (Fig. 4a, Table 1, Supplementary Table S1). By hypergeometric analysis the ‘epithelial proliferation’ (hP = 1.7E-21) and ‘epithelial differentiation’ (hP = 1.29E-16) clusters were highly significantly enriched for DE genes for diet conditions (Fig. 4a). The ‘metabolism’ cluster also showed enrichment for diet conditions DE genes (hP = 2.9E-04). A consistent pattern was found in the expanded epithelium network (Fig. 4b). The ‘epithelial proliferation-and-differentiation’ cluster was highly enriched for diet conditions DE genes (hP = 1.4E-42) and the ‘general metabolism’ cluster had a significant enrichment of diet conditions DE genes (hP = 1.5E-03). However, diet conditions DE genes were significantly under-represented in the ‘lipid/oxo-acid metabolism’ cluster (hP = 9.2E-08) (Fig. 2d). Although genes in this cluster were enriched for the GO-term “lipid metabolic process” (hP = 5.3E-5) (Fig. 2d), no diet conditions DE genes were found for the ketone body synthesis pathway (genes labelled in grey), a signature process of ruminant lipid metabolism^16^. DE genes for diet conditions were also significantly under-represented in the “muscle” sub-network (hP = 9.1E-15) (Fig. 4a). Diet conditions DE genes were not significantly over- or under-represented in the “type I interferon” sub-network.

The pattern of enrichment of diet conditions DE genes in the functional gene sets described above was also observed for transcription factors and their predicted targets (Fig. 4c). A majority of TFs related to ‘epithelial proliferation’ and ‘differentiation’, e.g., BRCA1, FOS, PRDM1, E2F4, GRHL1 and IRF6 and their targets were significantly enriched for diet conditions DE genes (hP = 8.1E-82) (Fig. 4c). The remaining epithelial TFs, mainly in the ‘metabolism’ cluster (Table 2 and Fig. 2) and including RXRA/PPARG, TP63/53, ELF3 and ZBTB33, were not significantly enriched for diet conditions DE genes (hP = 0.052). “Muscle” related TFs and their targets were significantly depleted for DE genes (hP = 4.2E-27) (Fig. 4c).

**Figure 5:**
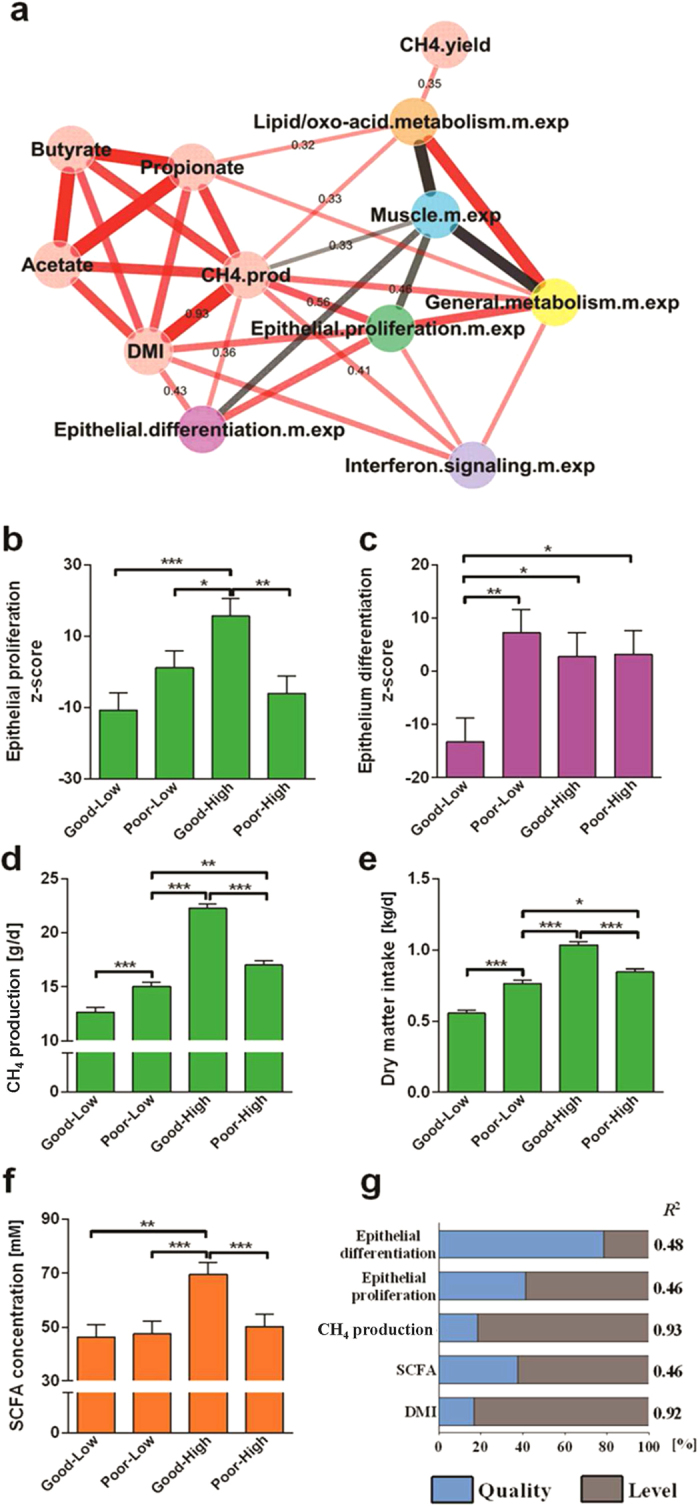
Relationships between expression of gene sets and phenotypes. (**a**) correlation between gene sets and phenotypes across the 24 samples determined by PCIT, which combines partial correlation and information theory to identify meaningful correlations, even though the value of the correlation coefficient may be low^48^. Edge width reflects the value of the correlation coefficient. Gene set colors correspond to the colors used in Fig. 2. Units for phenotypes values are: CH_4_.yield (g CH4/kg DMI), CH_4_.prod (g/d), DMI (kg/d) and concentration of acetate, butyrate and propionate (mM). Acetate, butyrate and propionate were measured in one sample/animal immediately following the measurement of methane production. Least square means of gene set expression and phenotypes vs. treatment groups were calculated based on full model for (**b**) ‘epithelial proliferation’ (cell cycle genes), (**c**) ‘epithelium differentiation’ genes, (**d**) CH_4_ production (g/d), (**e**) dry matter intake (kg/d) and (**f**) SCFA (acetate + propionate + butyrate) concentration (mM), sampled more than twelve hours after feeding. (**g**) Contributions of feed quality and level to gene set expression and phenotype within explained variations (R^2^).

The average Z-scores of the expression level of genes in the gene sets identified above (Supplementary Table S1) were used to determine their relationships with diet and CH_4_ phenotypes. The relationships between phenotypic measurements are stronger than the relationships between phenotypes and expression of gene sets (Fig. 5a). Overall, feed level explained the majority of the variation in all phenotypes and gene expression, except the ‘epithelial differentiation’ set where the majority of variation in the expression was explained by feed quality (Fig. 5b–g). The treatment group with the good quality and higher feed availability had the highest ‘epithelial proliferation’ gene expression levels and highest DMI and CH_4_ phenotypic values (Fig. 5a,d–f). This is consistent with the strong positive correlation-triangle between expression of the ‘epithelial proliferation’ set and DMI (kg/d) and CH_4_ production (g/d) (Fig. 5a). The variations in expression of ‘epithelial differentiation’ set was driven by the lowest expression in the good quality and low availability feed group (Fig. 5c,g). A positive correlation was observed between DMI (kg/d) and the expression of genes in the ‘epithelial differentiation’ set (Fig. 5a). The expression of the “lipid/oxo-acid metabolism’ gene set showed weak positive correlations with SCFA concentrations (sampled more than twelve hours after feeding) and with CH_4_ production (g/d) (Supplementary Table S4, Fig. 5a). The only meaningful, but weak, correlation between CH_4_ yield (g CH_4_ production/kg DMI) and expression level of any gene sets identified by PCIT was with the lipid/oxo-acid metabolism cluster (Fig. 5a, Supplementary Table S4).

## Discussion

In our study we have applied network approaches to extensively analyse sheep rumen transcriptomic data in parallel with bovine and human skin transcriptomes and in the context of diet and CH_4_ production (g/d). The sheep rumen gene expression correlation network mirrored the composition and activities of different parts of the rumen wall (Supplementary Figure S1). However, very few genes in the muscle-related subnetwork responded to dietary conditions, or were associated with CH_4_ production (g/d) or yield (g CH_4_ /kg DMI). Gene expression in the epithelial layers was much more responsive to diet and the most responsive gene cluster is enriched for genes expressed in progenitor keratinocytes, including cell cycle genes, and contains the widely used cell proliferation marker *MKI67* reported to be expressed in some, but not all, basal cells and some early suprabasal cells in human skin^24^. We interpret the increase in cell cycle gene expression as an increase in cell division in the transit amplifying cells in the basal layers due to increased turnover of the epithelium and/or increased epithelial volume (greater epithelial thickness and/or increased size/density of papillae) relative to muscle volume in response to increased intake^25^,^26^. Such changes would increase the proportion of epithelial cells, and hence expression of genes preferentially expressed in the epithelium, relative to the other predominantly muscle cells, and hence genes preferentially expressed in the muscle, in the full thickness rumen wall samples. In order to maintain a consistent thickness of epithelial layers; in general as the division rate of progenitor cells increases, so does the turnover of differentiated cells^27^. The close correlation of the expression of genes in the epithelial proliferation and differentiation clusters (Figs 1a and 2a) is consistent with this model. However, the relationship between diet and the rumen epithelium is complex, a positive relationship between cell division (mitotic index) and papillae length/number has been reported in the rumen of both sheep^28^ and cattle^29^, and a negative relationship between cell division/papillae density and the thickness of the epithelial layers has also been reported in both sheep^28^,^30^ and cattle^29^. Our results are consistent with increased papillae length/number and hence mitotic index on higher quality diets and with dietary conditions being a driver of variation in cell cycle gene expression. Both increased and decreased keratinization has been reported for poor/rough diets^30^,^31^,^32^,^33^. Again our identification of dietary conditions as a driver of variation in expression of epithelial differentiation genes is consistent with these observations. It is thought that the availability of SCFAs, in particular butyrate, plays a role in the control of the amount of epithelium per unit area of the rumen muscle layers (discussed in ref. 34). Our observations of positive correlations between the expression of metabolism genes and SCFA concentrations (sampled more than twelve hours after feeding) (Supplementary Table S4, Fig. 5a) are consistent with this hypothesis, but do not prove it.

In addition to the closely linked epithelial ‘proliferation’ and ‘differentiation’ gene sets the expanded epithelial network contained two other major clusters (Fig. 2a). One major cluster is the ‘lipid/oxo-acid metabolism’ cluster containing two gene sets: additional epithelial genes (purple) and genes significantly enriched for lipid/oxo-acid metabolism processes (olive and brown) (Fig. 2a,d), as identified in the global network (Fig. 1a). The metabolic activity of the rumen wall is thought to be highest in the basal cells of the epithelium, which have the highest density of mitochondria^35^. Consistent with this the SCFA transporter *SLC16A1* (aka *MCT1*) and the cation proton antiporter *SLC9A2* (aka *NHE2*), located in the ‘lipid/oxo-acid’ metabolism cluster, have been shown to be highly preferentially expressed in the basal layer of the bovine rumen^36^. Thus the ‘lipid/oxo-acid’ metabolism cluster may represent the energy acquisition processes of predominantly the basal cells of the epithelium. Genes responding to diet are actually under-represented in this cluster (Fig. 4b) suggesting that at the global level expression of rumen metabolic genes (or lipid/oxo-acid metabolic pathways) and/or numbers of basal cells is not significantly affected by the dietary differences imposed in this experiment. The additional epithelial gene set (purple) in the ‘lipid/oxo-acid’ metabolism cluster also contains a small number of genes associated with ruminant specific epidermal late differentiation (e.g., *TCHHL2* and *IVL*, Fig. 2a)^17^. This set of genes has a slightly different expression profile from the majority of the late differentiation genes (e.g., *S100A* and *SPRR2-like* gene family members, Fig. 2a) in the ‘epithelial proliferation-and-differentiation’ cluster.

The ‘general metabolism’ genes are in the other major cluster in the expanded epithelial gene subnetwork that contains genes with roles with metabolic activity. However, these genes encode proteins involved in more general cellular processes (electron transport and intracellular transport (yellow and light orange, Fig. 2d,e) important in all cells. This cluster probably represents the integration of the metabolic activity of all of the epithelial layers of the rumen wall. Although late stage keratinocyte differentiation genes were under-represented in this cluster (Fig. 2c), four members of the ruminant-specific *PRD-SPRRII* family located in the EDC region (Fig. 2a) were present. These genes have evolved from an *SPRR2*-family gene at the base of the ruminants^5^. In contrast those members of the family most similar to the *SPPR2* progenitor (the *SPRR2* and *SPRR2*-like genes) are located in the ‘epithelial proliferation-and-differentiation’ cluster. Their expression pattern suggests that the more recently evolved *PRD-SPRRII* family may have a different regulation and function from the older *SPRR2* family members. The separation of EDC locus genes critical for epithelial differentiation and the separation of ‘general’ and ‘lipid/oxo-acid’ metabolism and ‘differentiation’ clusters in the rumen epithelial network implies the existence of specific regulatory processes defining different components of the rumen wall metabolic and cornification processes, perhaps with different emphases in different cell types. Unlike the TFs associated with epithelial proliferation and differentiation, the TFs in the metabolic gene clusters were less clearly associated with expression in basal or suprabasal human skin (Table 2). Probably because metabolism is a much less cell type specific process (limited enrichment of late differentiation markers in any of these clusters, especially the general metabolism, Fig. 2c), than differentiation. However, overall it shows that in this study genes involved in ‘general processes’ rather than genes involved in ‘lipid/oxo-acid processes’ were most responsive to different diet conditions, consistent with the apparent increased turnover/amount of the epithelium. In addition, potential regulators of the general and lipid/oxo-acid metabolic processes in the rumen wall have been identified (Table 2).

Our analyses do not provide an answer to the question of what the cellular origin of the genes in the “Type 1 interferon” sub-network is. The expression of the genes is not significantly correlated with the muscle sub-network, or to any of the clusters in the epithelium sub-network. In addition genes whose expression is affected by diet are neither over nor under-represented in the sub-network, although their expression varies between animals. Currently the most likely explanation appears to be a transient population of cells in the rumen wall, perhaps with an immune system function. However, further investigation of the origin of this cluster of genes is required.

Our identification of common transcription factors between human skin and sheep rumen suggests a conservation of epithelial growth control systems. Thus insights into the growth and control of development, proliferation and differentiation of the epithelial layers of the rumen will be gained by further comparative study with skin. However, there are also likely to be rumen specific aspects to these processes. Since rumen epithelial features are closely linked to feed intake and digestion^10^,^11^,^12^, not characteristics of skin, understanding the regulatory systems controlling the change of the rumen epithelial growth in response to changes in the environment will enable better management of ruminant digestion. In this work we have identified a number of candidates for further investigation (Table 2).

CH_4_ production is a consequence of complex interactions between the rumen wall, diet and local microbes and is associated with gastrointestinal digestion and energy production efficiency^37^. Consistent with the literature^38^, in our experiment CH_4_ production was primarily determined by DMI (Fig. 5a). We found a stronger relationship between diet conditions and rumen epithelial proliferation and differentiation, than between diet conditions and the metabolic and muscle systems. This led to the strong relationship between CH_4_ production (g/d) and DMI and epithelial proliferation and differentiation. This is consistent with the previous evidence where variations in CH_4_ emissions were associated with the changes in the rumen size^39^. CH_4_ yield (g CH_4_/kg DMI) is a complex phenotype with low heritability^15^ and the host genetic influences were evident in a large scale industry breeding program^13^. CH_4_ yield (g CH_4_/kg DMI) is not correlated with CH_4_ production (g/d) (Fig. 5a), but we detected a weak relationship between the expression of genes in the lipid/oxo-acid metabolism cluster, which included genes in the ketone-body synthesis pathway^16^, and both CH_4_ yield (g CH_4_/kg DMI) and production (g/d). These correlations, which appear to be biologically sensible, are weak, potentially due to the small number of animals studied, the use of single time point measurements of [SCFA], the time gap between measurements and the sampling of the animals for tissue samples for transcriptomic analysis and a complex relationship between expression of genes in the rumen wall and the microbial processes responsible for CH_4_ production. In addition, correlations do not identify cause and effect, only generate hypotheses to be tested. Access to datasets from larger and/or other independent experiments are required to reliably increase our understanding of the relationships between the host rumen functional gene sets and CH_4_ production and yield, etc. Our current analysis has laid the foundation for significantly increased understanding of the control of rumen epithelial development and function, and has identified candidate molecular mechanisms by which the animal may influence CH_4_ emission from the contents of the rumen.

## Materials and Methods

### Animal experiment

The animal experiments were reviewed and approved by the Grasslands Animal Ethics Committee (Palmerston North, New Zealand approval #13146) and animals were cared for according the New Zealand Code of Ethical Conduct (Animal Welfare Act, 1999) and its amendments. All methods were carried out in accordance with the approved guidelines. Twenty four 8–9 months old female sheep were selected from a methane experiment^2^,^15^ at Grasslands Research Centre (AgResearch Ltd. Palmerston North, New Zealand) in May 2014. Animals were randomly assigned to one of the four dietary treatments (six animals per treatment): freshly cut forage at two qualities, vegetative growth [good] and mature [poor] (Supplementary Table S5), and two feeding levels ~1.0 × [low] and 1.8 × [high] (effectively *ad libitum* for the low quality diet) of metabolisable energy for maintenance based on the weight of the animals. Pastures dominated by perennial ryegrass at vegetative and mature stages were cut daily around 11 am. Cut pasture was subdivided into two equal portions for each treatment and stored in a chiller till feeding at ~3.30 pm and ~8.30 am the next morning. A representative sample was taken from each daily pasture cut and triplicate ~200 g subsamples were dried at 105 °C for 24 h to determine the dry matter (DM) content. During the measurement periods, another subsample was dried at 65 °C for 48 h, ground to pass through a 1 mm screen, and analysed by the Nutrition Laboratory of Massey University (Palmerston North) for DM, ash, nitrogen (N), gross energy (GE), neutral detergent fibre (NDF), acid detergent fibre (ADF), acid detergent lignin (ADL) and *in vitro* cellulase digestibility according to Roughan and Holland^40^.

Feed intake and CH_4_ emissions were measured over two consecutive days in individual open-circuit respiration chambers at the New Zealand Ruminant Methane Measurement Centre^41^ with feeding as above. Animals were first acclimatized in pens and then in metabolic crates, followed by measurements of gaseous emissions in respiration chambers (Supplementary Table S6). Animals were offered feed based on their weight and treatment group (see above), uneaten feed was weighted to calculate actual DMI intake. After sheep were removed from the chambers, rumen contents were collected by stomach tube immediately prior to the next morning feed for SCFA analyses including acetate, propionate, butyrate by gas chromatography^42^,^44^. The animals were slaughtered over two days using captive bolt. Animals were fed ~2 hrs prior to slaughter. Full thickness rumen wall samples, 2 × 1 g, were taken after slaughter at equivalent sites in the ventral part of the rumen atrium of all the animals, and snap-frozen in liquid N_2_ and stored at −80 °C for RNA extraction.

### RNA preparation and sequencing

The whole full thickness rumen wall samples were shipped to CSIRO’s FD McMaster Laboratory at Chiswick, Armidale, Australia and transferred into RNALater^®^-ICE Frozen Tissue Transition Solution (Ambion^®^) and stored at −20 °C. RNA was extracted from the full thickness of ~200 mg of ventral rumen tissue using the Qiagen RNeasy^®^ Midi Kit with on-column DNase digestion. The average RNA concentration measured using a Quant-iT™ RiboGreen^®^ RNA reagent and kit (Invitrogen™) on a fluorescent plate reader (excitation 485 nm and emission 538 nm) was 333.9 ± 143.4 (SD) ng/μl. Samples of ~4 μg of total RNA were rRNA depleted using the Ribo-Zero™ Magnetic Gold Kit (Human/Mouse/Rat) (Epicentre^®^) and purified using a Qiagen RNeasy^®^ MinElute Cleanup Kit. 12 μl rRNA depleted RNA was sent to the Ramaciotti Genomics Centre, The University of New South Wales, Australia. RNA Quality was checked and stranded libraries were prepared using SureSelect™ stranded RNA sample preparation kit (Agilent Technologies) at the sequencing centre. RNA sequencing was performed as 100 base paired-end (PE) reads in two lanes of an Illumina HiSeq2000 instrument housed at the Ramaciotti Genomics Centre. Total reads per sample ranged from 15–20 (single reads) or 30–40 million (PE) per sample. RNA sequencing results were checked for quality using FastQC at the sequencing center.

### Data analyses

RNA sequencing raw reads files (fastq) of the full thickness rumen wall transcriptome were retrieved from the sequencing Centre. Raw reads were aligned to Ensembl sheep gene models v3.2 with additional models for genes at the EDC locus [14] which is not well annotated in the Ensembl sheep gene models, using tophat^44^ and bowtie2^45^ with default options. Generated BAM files were calculated for counts using HTSeq in the Python environment^46^. A global gene network was constructed with VOOM^47^ and PCIT^48^ in R v3.1.3 based on VOOM^47^ normalised gene expression values of transcripts with at least 3-counts per million in all 24 rumen samples. The PCIT output was filtered for pairs of genes with a correlation coefficient >+0.8 visualized in Cytoscape v3.1.2^49^. The expanded epithelium network was built with the same gene input as the global network, but without including the genes clustered in the ‘muscle’ and ‘type I interferon’ related sub-networks. A lower correlation coefficient threshold (>+0.75) was used to build the epithelium network and the Glay community clustering plugin in Cytoscape was used to subdivide the network into clusters (Fig. 2). Sets of genes were mapped to the networks using standard procedures in Cytoscape.

We performed a cross-species transcriptomic meta-analysis to compare the expression level of genes in the full thickness rumen wall, rumen papillae and smooth muscle. We retrieved transcriptomic data from a bovine rumen papillae RNA sequencing experiment^18^ and converted SRA files to fastq files using the SRA Toolkit (http://www.ncbi.nlm.nih.gov/books/NBK158900/). Mouse small intestinal smooth muscle microarray transcriptomic data^19^ was also retrieved. Bovine rumen papillae BAM files were build using the procedures described above with the latest bovine gene models in the UMD3.1 genome assembly from Ensembl and count data was obtained by HTSeq^46^. For sheep genes with at least 1 count per million in at least one of the 24 rumen samples, their bovine and mouse orthologs were mapped and the best mapping was determined by the highest identity percentage (Supplementary Table S2). Mapped bovine rumen papillae gene count data was transformed by VOOM^47^ and mouse smooth muscle gene expression data was log2 transformed. Transformed bovine and mouse data were jointly normalised with sheep full thickness rumen wall gene VOOM transformed values using the quantile method^50^. Normalised gene expression values were tested for three comparisons: full thickness rumen wall vs. rumen papillae (Fig. 1b), smooth muscle vs. rumen papillae (Supplementary Figure S2a) and smooth muscle vs. full thickness rumen wall (Supplementary Figure S2b). These comparisons were tested for differential expression and log2 fold change using limma^51^ (Supplementary Table S1). Differentially expressed genes (P and FDR <0.05 with absolute log_2_ fold change values >1) in the comparison of rumen papillae vs. full thickness rumen wall were mapped to the general sheep gene network described above (Fig. 1b). Distribution box plots of fold change for gene clusters were visualized using the ggplot_boxplot function^52^ in R.

The iRegulon^53^ plugin in Cytoscape was used to identify motif enriched (normalized enrichment score >=3) transcription factors (Fig. 3) in the “epithelium”, “muscle” and “type I interferon” sub-networks in the general network and differentially expressed for diet conditions. Gene sets were mapped to the transcription factor network using standard Cytoscape procedures. We analysed and compared rumen identified transcription factors’ expression pattern in human skin using Genevestigator^54^. A total of 1460 samples of human microarray data in the Genevestigator database generated from skin samples, or skin derived cell lines, were retrieved for the identified transcription factors. The data including cell lines of all skin layers including basal, suprabasal and epidermal keratinocytes was analysed by hierarchical clustering. The same approach was applied to investigate EDC locus^5^ and keratin genes.

Differentially expressed genes for diet conditions in this experiment were analysed as follows. After filtering for transcripts with at least 1 count per million in at least one of the 24 rumen samples, data was analysed using the Analysis of Variance-like procedure (EdgeR) in R using the model *y* = *quality*_*level* + *e*_*ij*_. The *quality*_*level* variable has four levels representing the combination of feed quality and level variables: Good-High, Good-Low, Poor-High, Poor-Low. Since the analysis was undertaken to identify enrichment in clusters rather than specific genes, genes with raw P values < 0.05 were selected and mapped to the high stringency-filtered global gene. The probability of over, or under, representation of differentially expressed genes for diet conditions in the global gene network was calculated using the hypergeometric distribution (hP)^55^ for 5 major gene sub-networks and clusters (muscle, cell cycle, metabolism, epithelial differentiation and type I interferon, Fig. 4a). Functional enrichment of gene sub-networks and clusters was analyzed using GOrilla^56^ to identify biological pathways and only pathways with FDR adjusted enrichment eP values < 0.05 were taken into consideration (Fig. 1a).

Phenotypic data and Z-score of defined gene sets (described below) were analysed using general linear models with stepwise backwards elimination^57^,^58^. Variables fitted in the initiated model included main effects, i.e., feed quality (good/poor), feed level (high/low) and all two way interactions. Only significant variables were retained in the final model after the stepwise variable elimination (Fig. 5, Supplementary Table S3). Contributions of feed quality and level to variations in phenotypic and gene set expression using type 1 sum of square values followed previous procedures^57^,^59^. The Z-score of a gene set was estimated as the mean of Z-scores of FPKM of each gene member. Correlation coefficient based networks of gene sets and phenotypes were built using PCIT^48^ in R and presented in Cytoscape with the same procedures as above. All calculated correlation coefficients and their significance levels are presented in Supplementary Table S4.

### Data Availability

Raw sequence data generated in this study has been submitted to the National Center for Biotechnology Information Sequence Read Archive (SRA; http://www.ncbi.nlm.nih.gov/sra/) under accession no. PRJNA313132.

## Additional Information

**How to cite this article**: Xiang, R. *et al*. Gene network analysis identifies rumen epithelial cell proliferation, differentiation and metabolic pathways perturbed by diet and correlated with methane production. *Sci. Rep.* **6**, 39022; doi: 10.1038/srep39022 (2016).

**Publisher's note:** Springer Nature remains neutral with regard to jurisdictional claims in published maps and institutional affiliations.

## Supplementary Material

Supplementary Information

Supplementary Tables

## Figures and Tables

**Table 1:** Summary of enrichment analyses.

Sub-network				Cluster	Differentially expressed genes in network clusters					
	Enriched gene sets in sub-networks				Full thickness rumen wall vs rumen papillae		Progenitor vs late differentiation skin keratinocytes		Diet effects	
	GO term/pathways	Gene number	eP^1^		Gene number^2^	hP^3^	hP^4^ progenitor	hP^5^ late differentiation	hP^6^ global network	hP^6^ epithelium network
Muscle	Extracellular matrix organization (0030198)	84	4.7e-23	Muscle	71↑	3.2e-74 (over)	not tested	not tested	9.1e-15 (under)	not tested
	Cell motility (0048870)	78	2.3e-08		68↑					
	Muscle system process (0003012)	38	2.2e-05		34↑					
	Regulation of DNA-templated transcription (0006355)	235	1.5e-05		175↑					
Type 1 interferon	Type 1 interferon signalling pathway (0060337)	6	3.3e-05	Type 1 interferon	4↑	1.2e-01 (n.s.)	not tested	not tested	1.8e-01 (n.s.)	not tested
Epithelium	Cell cycle (0007049)	44	9.2e-32	Epithelial proliferation	21↓	3.6e-09 (over)	2e-12 (over)	5.6e–02 (over)	1.7e-21 (over)	1.4e-42 (over)
	Epithelial differentiation^7^	16	2.0e-10	Epithelial differentiation	3↓	2.3e-01 (n.s.)			1.3e-16 (over)	
	Oxo-acid metabolic process (0043436)	27	2.1e-19	Lipid/oxo-acid metabolism	10↓	3.8e-44 (over)	5.1e-12 (under)	1.7e-02 (over)	2.9e-04 (over)	9.2e-08 (under)
	Lipid metabolic process (0006629)	46	4.6e-10		26↓					
	Intracellular transport (0046907)	60	3.1e-13	General metabolism	33↓		4.3e-10 (under)	1.5e-06 (under)		1.5e-03 (over)
	Respiratory electron transport chain (0022904)	17	2.5e-11		8↓					

^1^FDR Corrected enrichment eP value of GO-term.

^2^↑ and ↓ indicate higher and lower expression, respectively, in full thickness rumen wall compared to rumen papillae.

^3^hP-value hypergeometric test of representation of differential expression between full thickness rumen wall and rumen papillae with P and FDR <0.05 in the cluster. Over/under: over- or under- representation of genes in respect network for hypergeometric test. n.s.: not significant for hypergeometric test.

^4^hP-value for over or under representation of genes more highly expressed in progenitor cells relative to late differentiation cells in *in vitro* keratinocyte differentiation dataset^17^.

^5^hP-value for over or under representation of genes more highly expressed in late differentiation cells relative to progenitor cells in *in vitro* keratinocyte differentiation dataset^17^.

^6^hP-value hypergeometric test of representation of differential expressed genes with P < 0.05 for diet conditions (Supplementary Table S1) in the cluster. Over/under: over- or under- representation of genes in respect network for hypergeometric test. n.s.: not significant for hypergeometric test.

^7^Enrichment of EDC locus genes, not annotated as a group in GO.

**Table 2:** Summary of transcription regulatory factor and epidermal marker analyses.

Gene description	Name	Global network	Expanded epithelial network	Human skin expression^1^
Breast cancer 1	*BRCA1*	Cell cycle	EPD – CC^2^	basal epidermis
Hyaluronan-mediated motility receptor	*HMMR*	Cell cycle	EPD – CC	basal epidermis
Marker of proliferation Ki-67	*MKI67*	Cell cycle	EPD – CC	basal epidermis
Tumor protein p53	*TP53*	n/p^3^	n/p	basal epidermis
Enhancer of zeste 2 polycomb repressive complex 2 subunit	*EZH2*	n/p	EPD – CC	suprabasal epidermis
ets homologous factor	*EHF*	Epithelium differentiation	EPD – Diff	suprabasal epidermis
PR domain zinc finger protein 1	*PRDM1*	Epithelium differentiation	EPD – Diff	suprabasal epidermis
Grainyhead-like 3	*GRHL3*	Epithelium differentiation	EPD – Diff	suprabasal epidermis
Grainyhead-like 1	*GRHL1*	n/p	EPD – Diff	suprabasal epidermis
Ovo-like zinc finger 1	*OVOL1*	Epithelium differentiation	EPD – Diff	suprabasal epidermis
FBJ osteosarcoma oncogene	*FOS*	n/p	EPD – Diff	suprabasal epidermis
v-myc avian myelocytomatosis viral oncogene homolog	*MYC*	n/p	EPD – Diff	nd
E2F transcription factor family 4	*E2F4*	n/p	General metabolism	suprabasal epidermis
Zinc finger and BTB domain containing 33	*ZBTB33*	Metabolism	General metabolism	basal epidermis
Receptor interacting serine/threonine kinase 4	*RIPK4*	n/p	Lipid/oxo-acid metabolism	basal epidermis
Tumor protein p63	*TP63*	Metabolism	Lipid/oxo-acid metabolism	basal epidermis
Retinoid X receptor	*RXRA*	Metabolism	Lipid/oxo-acid metabolism	suprabasal epidermis
Zinc Finger Protein 750	*ZNF750*	Metabolism	Lipid/oxo-acid metabolism	suprabasal epidermis
E74-like factor 3 (ets domain transcription factor, epithelial-specific)	*ELF3*	Metabolism	Lipid/oxo-acid metabolism	nd
Peroxisome proliferator-activated receptor gamma	*PPARG*	Metabolism	Lipid/oxo-acid metabolism	nd
Interferon regulatory factor 6	*IRF6*	n/p	Lipid/oxo-acid metabolism	nd
Mitogen-activated protein kinase 1	*MAPK1*	n/p	n/p	suprabasal epidermis

^1^expression in basal v suprabasal epidermis data from Genevestigator^54^ (also shown in Fig. 2f), original data from Gulati *et al*. 2013. “nd” was not differentially expressed between basal epidermis and suprabasal epidermis.

^2^EPD – CC is Epithelial proliferation and differentiation, cell cycle sub-cluster.

^3^n/p: not present.

^4^EPD- Diff is Epithelial proliferation and differentiation, differentiation sub-cluster.
