# Supplementary material for: Gene network analysis identifies rumen epithelial cell proliferation, differentiation and metabolic pathways perturbed by diet and correlated with methane production
Source: Sci Rep. 2016 Dec 14;6:39022. doi: 10.1038/srep39022 (PMC5155297; doi:10.1038/srep39022)
Supplement: Supplementary Information [file srep39022-s1.pdf]

## Title page of Supplementary Figures S1-2 for:

Gene network analysis identifies rumen epithelial cell proliferation, differentiation and metabolic pathways perturbed by diet and correlated with methane production

Ruidong Xiang[1], Jody McNally[2], Suzanne Rowe[3], Arjan Jonker[4], Cesar S. Pinares-Patino[,4,5], V. Hutton Oddy[6], Phil E. Vercoe[7], John C. McEwan[3], Brian P. Dalrymple[1,7*]

[1]CSIRO Agriculture, Queensland Bioscience Precinct, 306 Carmody Rd, 4067 Queensland, Australia; [2]CSIRO Agriculture, FD McMaster Laboratory, Armidale, NSW 2350, Australia; [3]AgResearch Limited, Invermay Agricultural Centre, Mosgiel 9053, New Zealand; [4]AgResearch Limited, Grasslands Research Centre, Palmerston North 4442, New Zealand; [5]CSIRO Agriculture, Black Mountain Laboratories, Clunies Ross Street, 2601 ACT, Australia; [6]NSW Department of Primary Industries, Beef Industry Centre, University of New England, Armidale, NSW 2351, Australia; [7]School of Animal Biology and Institute of Agriculture, The University of Western Australia, 35 Stirling Highway, Crawley WA 6009, Australia.

*Corresponding author: Brian P. Dalrymple, brian.dalrymple@csiro.au, brian.dalrymple@uwa.edu.au

# Supplementary Figure S1

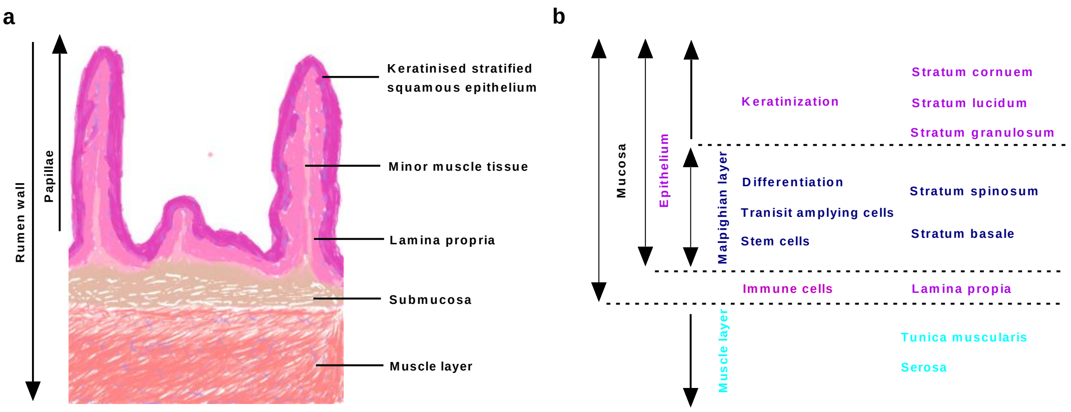


**Figure S1.** Organization of the rumen wall. (a) A diagrammatic representation of the rumen wall based on sheep rumen histology [1]. (b) Cell layers and their characteristics and roles in the epidermal differentiation process [2].

## Supplementary Figure S2

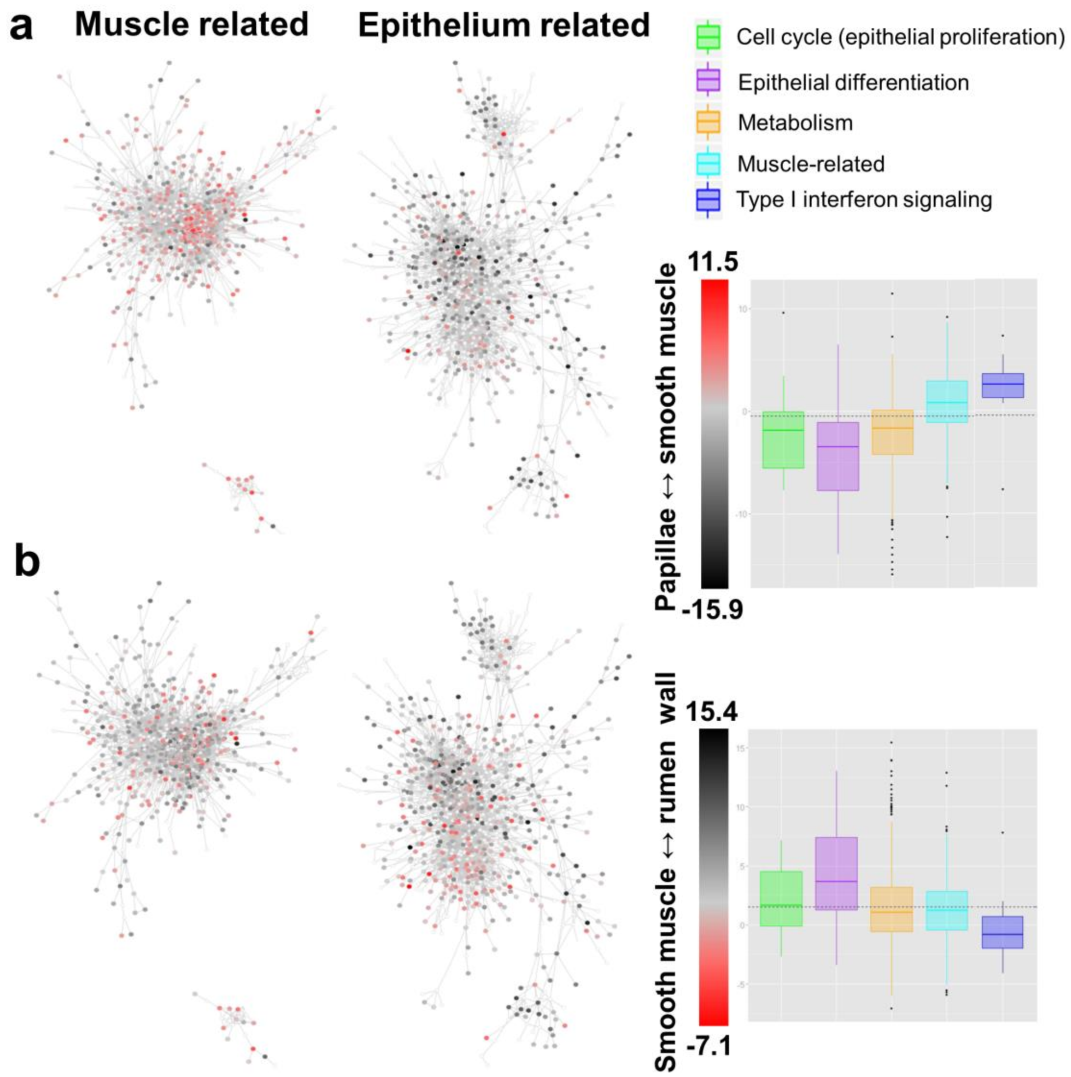


Figure S2. The global network with the results of transcriptomic comparisons mapped, **(a)** papillae vs smooth muscle and **(b)** smooth muscle vs full thickness rumen wall. The gradient from red to black represents log2 fold changes from high expression in the smooth muscle to high expression in the papillae **(a)** and high expression in the smooth muscle to high expression in the full thickness rumen wall **(b)**, with significant differential expression (P and FDR <0.05). Distribution box plots of log2 fold changes of expression of identified gene clusters for each comparison are also presented.

## References:


1. Dobson MJ, Brown WC, Dobson A, Phillipson AT. A histological study of the organization of the rumen epithelium of sheep. *Q J Exp Physiol Cogn Med Sci*. **41**, 247-53 (1956).
2. Hsu Y-C, Li L, Fuchs E. Emerging interactions between skin stem cells and their niches. *Nat Med*. **20**, 847-56 (2014).
